# Confessions of a Disease Monger

**DOI:** 10.1371/journal.pmed.0030314

**Published:** 2006-07-25

**Authors:** James Phelps

**Affiliations:** **1**Co-Psych.com, PsychEducation.org

I am a disease monger. I teach primary care doctors how to identify bipolar disorder. Worse yet, I take money from pharmaceutical companies for doing so. I use it to subsidize my practice so that I can treat patients with no insurance, or little money, who now account for over a third of my patients—in part because the pharmaceutical companies have drained so much money out of the health-care system. Ironic, isn't it?

My Web site PsychEducation.org ( http://www.psycheducation.org) is number one on Google for searches on “bipolar II.” See if you think it looks like disease mongering. Hundreds of people have written thanking me for explaining bipolar II and the concept of a bipolar spectrum, indicating that this new perspective really helps them understand their long-standing symptoms. To my immediate recall, none have complained about being led astray by an overbroad interpretation of bipolarity.

Notice that, just like Mr. Moynihan, one of the guest editors of your April 2006 series of articles on disease mongering [ 1], I could be mongering even now, as I too have a new book. At least I'm not trying to attract attendees to my conference. Tricky, isn't it. Has there been an oversimplification in this analysis?

(PsychEducation.org earned an Honorable Mention Moffic Award for Ethical Practice in Community Psychiatry, 2005.)
